# SIRT1 activation by minocycline on regulation of microglial polarization homeostasis

**DOI:** 10.18632/aging.103542

**Published:** 2020-09-23

**Authors:** Ling-Hsuan Wu, Bor-Ren Huang, Sheng-Wei Lai, Chingju Lin, Hsiao-Yun Lin, Liang-Yo Yang, Dah-Yuu Lu

**Affiliations:** 1 Graduate Institute of Basic Medical Science, China Medical University, Taichung, Taiwan; 2 Department of Neurosurgery, Taichung Tzu Chi Hospital, Buddhist Tzu Chi Medical Foundation, Taichung, Taiwan; 3 School of Medicine, Tzu Chi University, Hualien, Taiwan; 4 Department of Physiology, School of Medicine, China Medical University, Taichung, Taiwan; 5 Department of Pharmacology, School of Medicine, China Medical University, Taichung, Taiwan; 6 Laboratory for Neural Repair, China Medical University Hospital, Taichung, Taiwan; 7 Biomedical Technology R&D Center, China Medical University Hospital, Taichung, Taiwan; 8 Department of Photonics and Communication Engineering, Asia University, Taichung, Taiwan

**Keywords:** microglia, SIRT1, minocycline, microglial polarization, acetyl-p53

## Abstract

Sirtuin 1 (SIRT1) has been reported to be involved in the mechanisms underlying longevity and has also been indicated as a valuable regulator of age-related neurological disorders. Some natural products increase SIRT1 activity and stimulate deacetylation of various proteins. In the present study, SIRT1 overexpression by genetic modification or treatment with SIRT1 activators significantly inhibited the secretion of nitric oxide and expression of inducible nitric oxide synthase, cyclooxygenase 2, and proinflammatory mediator—interleukin 1β—in microglia. SIRT1 activation also decreased the levels of K379 acetyl-p53 and the protein inhibitor of activated Stat 1 expression in microglial cells. In addition, it dramatically promoted M2 polarization of microglia, which enhanced cell motility and altered phagocytic ability. We also used minocycline, a well-known inhibitor of microglial activation, to study the mechanism of SIRT1 signaling. Minocycline treatment decreased neuroinflammatory responses and promoted M2 polarization of microglia. It also reduced the acetyl-p53 level in the brain tissues in an inflammatory mouse model. Our findings demonstrated that SIRT1 participates in the maintenance of microglial polarization homeostasis and that minocycline exerts regulatory effects on SIRT1 activation. Therefore, our results indicate that SIRT1 activation may be a useful therapeutic target for the treatment of neuroinflammation-associated disorders.

## INTRODUCTION

Microglial cells, which reside in the central nervous system, remove accumulating debris from the brain and are pivotal for maintaining tissue homeostasis, neuronal integrity, and network functioning [1]. Moreover, microglial activation triggers neuroinflammatory reactions and boosts neuronal death, which are the main pathological features of various neurodegenerative diseases [2, 3]. In addition, microglial activation and the expression of inflammatory mediators in the brain, including cytokines and chemokines, are considered key events in the pathogenesis of mood and cognition dysfunction [4, 5]. Upon stimulation, microglia change morphologically to larger ameboid cells and could be polarized to an inflammatory or anti-inflammatory phenotype under the influence of either a proinflammatory or anti-inflammatory microenvironment, designated as M1- or M2-activated microglia, respectively [3, 6]. Activated M1-polarized microglia can produce proinflammatory mediators such as inducible nitric oxide (NO) synthase (iNOS), cyclooxygenase 2 (COX-2), proinflammatory cytokines, and elevated secretions of neurotoxic factors, and thus contribute to brain inflammation, which might lead to neuronal degeneration [3, 5]. By contrast, the activation of M2-polarized microglia can produce anti-inflammatory mediators such as interleukin (IL)-4, IL-13, arginase 1 (ARG1), and chitinase-like-3 (Ym-1); stimulate secretions of neurotrophic factors to inhibit inflammation; and facilitate cell migration and phagocytic activity to clean debris [3, 5]. Development of compounds to modulate the shift of M1/M2 phenotypes has been suggested as a useful therapeutic strategy for neurological and psychiatric disorders with inflammatory components [7, 8].

The expression of heme oxygenase (HO)-1, an oxidative and cytoprotective enzyme, is upregulated during oxidative stress, cellular injury, and diseases [9–11]. Induction of HO-1 expression exerts an anti-inflammatory effect on macrophages [12–14]. HO-1 activation is considered as a potential therapeutic target for neuroinflammation and neurodegenerative diseases [9]. We previously reported that activation of the endogenous antioxidative enzyme, HO-1, exerts an anti-neuroinflammatory effect on microglial cells [15–18] and astrocytes [19], and that increased HO-1 expression protects neurons against neurotoxin-induced cell death [20]. In addition, early evidence [21] and our previous report [16] showed that enhancement of HO-1 expression can polarize macrophage/microglia toward the M2 phenotype.

SIRT1 (Sirtuin 1), also called the silent information regulator 2 protein, belongs to the sirtuin family of class III histone deacetylases (HDAC) and has recently been implicated in age-related diseases, including metabolic, cardiovascular, and neurodegenerative diseases [22]. Studies on resveratrol, a natural polyphenol that activates SIRT1, have implicated SIRT1 as a key regulator of energy and metabolic homeostasis [23, 24]. Our previous study also indicated that melatonin increased the SIRT1 expression level in glioblastoma, and further reduced the expression of cell adhesion molecules [25]. Emerging evidence suggests that SIRT1 plays a critical role in alleviating microglial activity. SIRT1 overexpression in microglia protected cells against Aβ toxicity in primary cortical cultures [26]. Moreover, SIRT1 deficiency in microglia contributes to cognitive decline in aging and neurodegeneration [27, 28]. SIRT1 has been reported to modify several transcriptional factors such as forkhead box O 1, p53, and nuclear factor κB [29–31]. Upregulated expression of p53 has been observed in both neurons and microglia in the brains of patients with Alzheimer disease (AD) [32, 33] and is associated with tau phosphorylation [34]. In addition, increased transcription-dependent p53 activity in microglia has been associated with the secretion of inflammatory cytokines and synaptic degeneration in neurons [35]. Overall, SIRT1 may be a useful therapeutic target for microglial neuroinflammation-associated disorders.

Minocycline, a broad-spectrum tetracycline antibiotic, is a highly lipophilic molecule with antioxidant and neuroprotective activities [36, 37]. Minocycline easily crosses the blood-brain barrier and is a specific microglial inhibitor [38]. Results of both laboratory and clinical studies show that minocycline exerts its anti-inflammatory actions by modulating microglial activation [39, 40] and the subsequent release of cytokines and chemokines [41–43], lipid mediators of inflammation [44], and NO release [45]; thus, it reduces the transcription of proinflammatory mediators in many neurological disorders [46–49]. In this study, we used minocycline to further evaluate the regulatory mechanisms of neuroinflammation and microglial polarization in SIRT1 activation.

## RESULTS

### SIRT1 activation suppresses proinflammatory cytokine expressions in microglial cells

**Figure 1:**
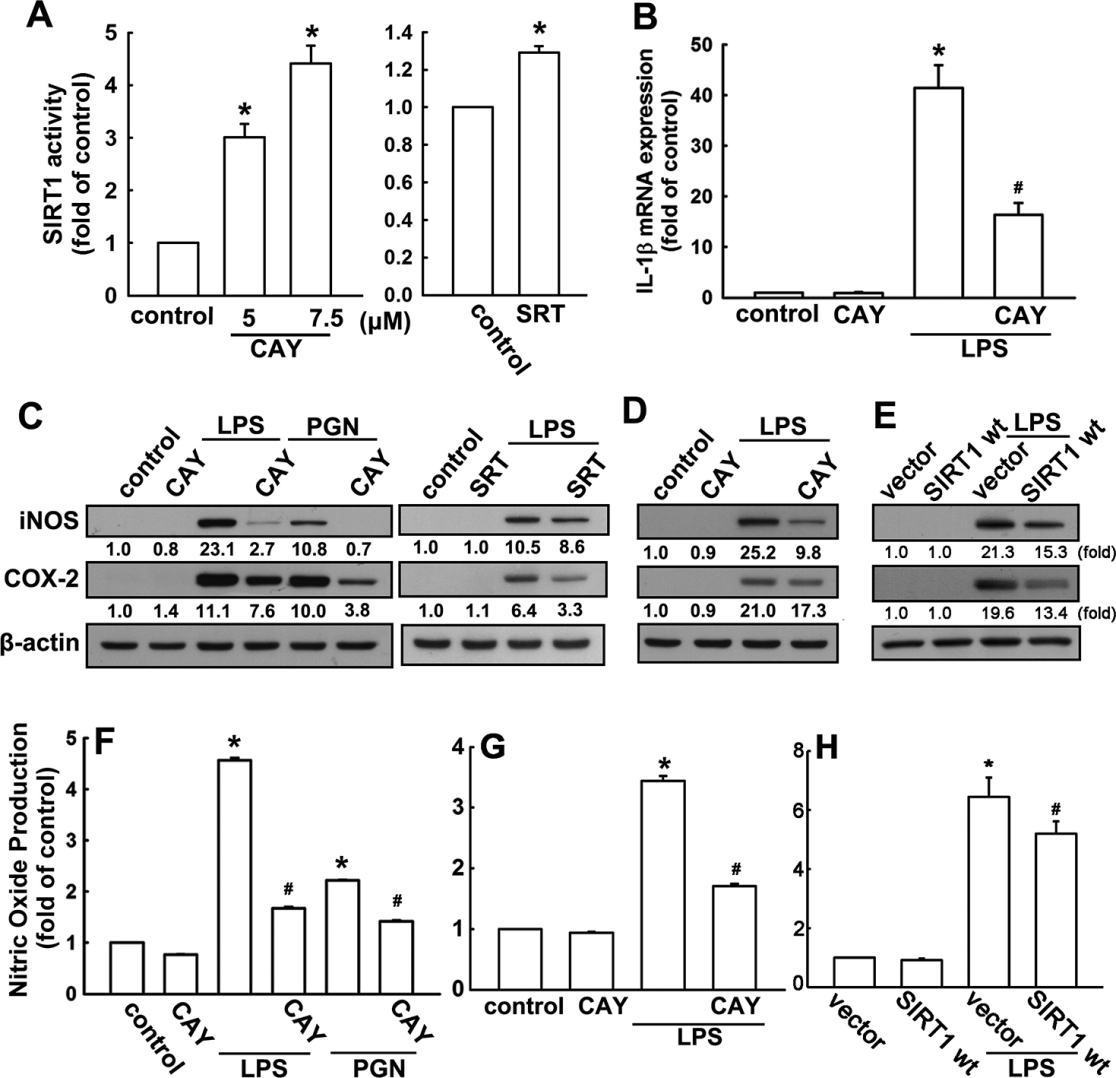
**Activation of SIRT1 suppresses neuroinflammatory responses in microglia.** (**A**) BV-2 microglial cells were incubated with various concentrations (5 or 7.5 μM) of SIRT1 activator (CAY) or SRT1720 (SRT; 1 μM)—for 24 h. Whole-cell lysate proteins were extracted and assessed using a fluorogenic SIRT1 assay kit to detect SIRT1 activity. (**B**) BV-2 microglial cells were pretreated with SIRT1 activator (5 μM) for 30 min, followed by stimulation with LPS (100 ng·mL^−1^) for 6 h. Relative mRNA levels of IL-1β were analyzed by real-time PCR and normalized with the levels of β-actin mRNA. (**C**) BV-2 microglia were pretreated with SIRT1 activator CAY compound (5 μM) or SRT (1 μM) for 30 min before stimulation with either LPS (100 ng·mL^−1^) or PGN (10 μg·mL^−1^) for 24 h. (**D**) Adult mouse microglia (IMG) were pretreated with CAY compound (5 μM) for 30 min before stimulation with LPS (100 ng·mL^−1^) for 24 h. (**E**) IMG cells were transfected with empty vector or wild-type SIRT1 for 24 h before stimulation with LPS (100 ng·mL^−1^) for 24 h. Whole-cell lysate protein was extracted and iNOS and COX-2 protein levels were assessed by western blot analysis. Culture media from BV-2 (**F**) or IMG (**G** and **H**) microglial cells were harvested to determine the nitrite content by the Griess reaction. The results represent the mean ± SEM of *n* = 3–4. * *p* < 0.05; compared with the control group, # *p* < 0.05; compared with LPS or PGN treatment groups.

The neonatal (BV-2) and adult (IMG) mouse microglial cells were used to study the anti-neuroinflammatory mechanisms of SIRT1 activation. BV-2 microglial cells stimulated with the SIRT1 activator, CAY compound, showed significant dose-dependent increase in SIRT1 activity (Figure 1A). In addition, cells treated with another SIRT1 activator, SRT1720 (SRT), further confirmed this phenomenon (Figure 1A). To determine the effect of SIRT1 activation on proinflammatory cytokine expression, cells were treated with a SIRT1 activator (CAY), followed by LPS or PGN stimulation. As shown in Figure 1B, treatment with CAY abrogated LPS-induced IL-1β expression. Moreover, administration of CAY significantly inhibited LPS- or PGN-induced iNOS and COX-2 expressions (Figure 1C). Treatment with another SIRT1 activator, SRT, also attenuated LPS-induced iNOS and COX-2 expressions (Figure 1C). In adult mouse microglial cells, such attenuation of LPS-induced iNOS and COX-2 expressions was mediated via SIRT1 activation (Figure 1D). Furthermore, SIRT1 overexpression effectively reduced the LPS-enhanced iNOS and COX-2 expressions in adult mouse microglia (Figure 1E). In addition, treatment with PGN and LPS increased NO production by approximately three- to six-fold. Moreover, the SIRT1 activator effectively antagonized LPS- or PGN-induced enhancement of NO production (Figure 1F). The attenuation effects of SIRT1 activation (Figure 1G) and overexpression (Figure 1H) were also observed in adult mouse microglial cells. In addition, SIRT1 activators at the concentration itself did not affect cell viability or NO production. These results suggest that SIRT1 activation effectively inhibits neuroinflammatory responses in microglial cells.

### SIRT1 activation decreases p53 acetylation and PIAS1 expression in microglial cells

**Figure 2:**
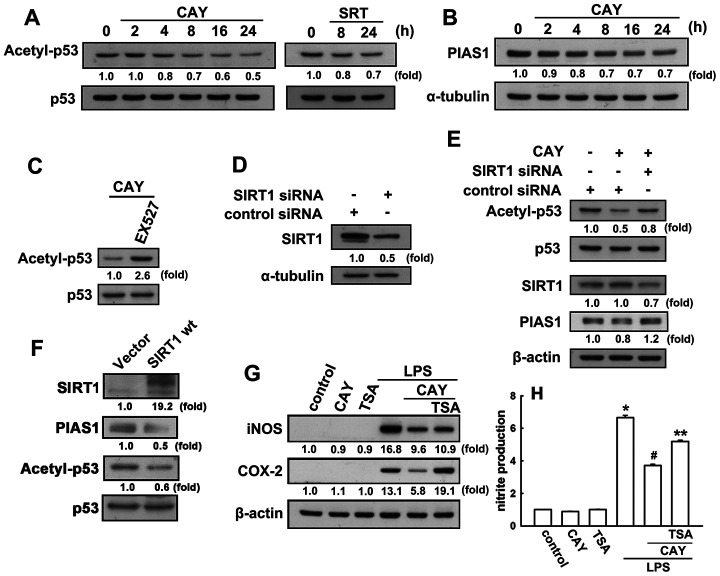
**SIRT1 decreases lysine 379 acetylation of p53 in microglial cells.** (**A**) BV-2 microglia were stimulated with CAY (5 μM) or SRT (1 μM) for the indicated time periods. The expression of K379 acetylated p53 was determined by western blot analysis. (**Β**) Microglial cells were stimulated with the SIRT1 activator, CAY (5 μM), for the indicated time periods (2–24 h). The expression of PIAS1 was determined by western blot analysis. (**C**) Microglial cells were treated with 10 μM EX527 (a SIRT1 inhibitor) for 30 min, followed by treatment with the SIRT1 activator, CAY (5 μM), for 24 h. Whole-cell lysate proteins were extracted and subjected to western blot analysis to assess lysine 379 acetylated p53. (**D**) After transfection of microglial cells with siRNA against SIRT1 or control for 24 h, the cells were lysed, proteins were extracted and the subjected to western blot analysis to assess SIRT1 expression. (**E**) Microglial cells were transfected with either siRNA against SIRT1 or control for 24 h, and then treated with CAY compound for another 24 h. Whole-cell lysate proteins were extracted and subjected to western blot analysis to assess acetylated p53, SIRT1, and PIAS1 expression. Similar results were obtained from at least three independent experiments. (**F**) IMG cells were transfected with empty vector or wild-type SIRT1 for 24 h, and the expression levels of SIRT1, PIAS1, acetyl-p53, and p53 were determined by western blot analysis. (**G**) After preincubation with TSA (10 nM) for 30 min, the SIRT1 activator, CAY (5 μM), was added for another 30 min before stimulation with LPS (100 ng·mL^−1^) for 24 h. The expression of iNOS and COX-2 was determined by western blot analysis, and the medium was collected to measure nitrite production (**H**). The results are presented as mean ± SEM of *n* = 3–4. * *p* < 0.05, compared with the control group; #, *p* < 0.05, compared with the LPS treatment group. **, *p* < 0.05 compared with the CAY plus LPS treatment group.

Next, we investigated whether SIRT1 activation leads to p53 deacetylation in microglial cells. When microglia were treated with a SIRT1 activator (CAY or SRT), a time-dependent decrease in p53 acetylation at lysine 379 levels was observed (Figure 2A). As shown in Figure 2C, EX527, a SIRT1 activity inhibitor, dramatically reversed the attenuation effect of the SIRT1 activator on the K379 acetylated p53. We then examined whether SIRT1 was involved in the p53 deacetylation in microglia. Transfection with SIRT1 siRNA abrogated SIRT1 expression (Figure 2D) and attenuated the acetyl-p53 levels in microglial cells (Figure 2E). Our previous study results have showed that PIAS1 is a regulator of the inflammatory process in microglia [50]. Treatment of microglial cells with CAY downregulated PIAS1 expression in a time-dependent manner (Figure 2B). Transfection with SIRT1 siRNA abrogated the inhibition of CAY-induced PIAS1 expression in microglia (Figure 2E). Moreover, transfection with wild-type SIRT1 increased SIRT1 levels but reduced the expression levels of PIAS1 and acetyl-p53 (Figure 2F). We further examined whether SIRT1 activation is required for anti-inflammatory responses in microglia. In the presence of trichostatin A, an HDAC inhibitor, the inhibitory effects of SIRT1 activation on LPS-induced iNOS and COX-2 expressions (Figure 2G) and nitrite production (Figure 2H) were alleviated. These results demonstrate that SIRT1 activation decreases PIAS1 expression level and deacetylates the K379 acetylated p53, which may participate in the regulation of inflammatory responses in microglia.

### SIRT1 activation promotes microglial M1/M2 polarization

**Figure 3:**
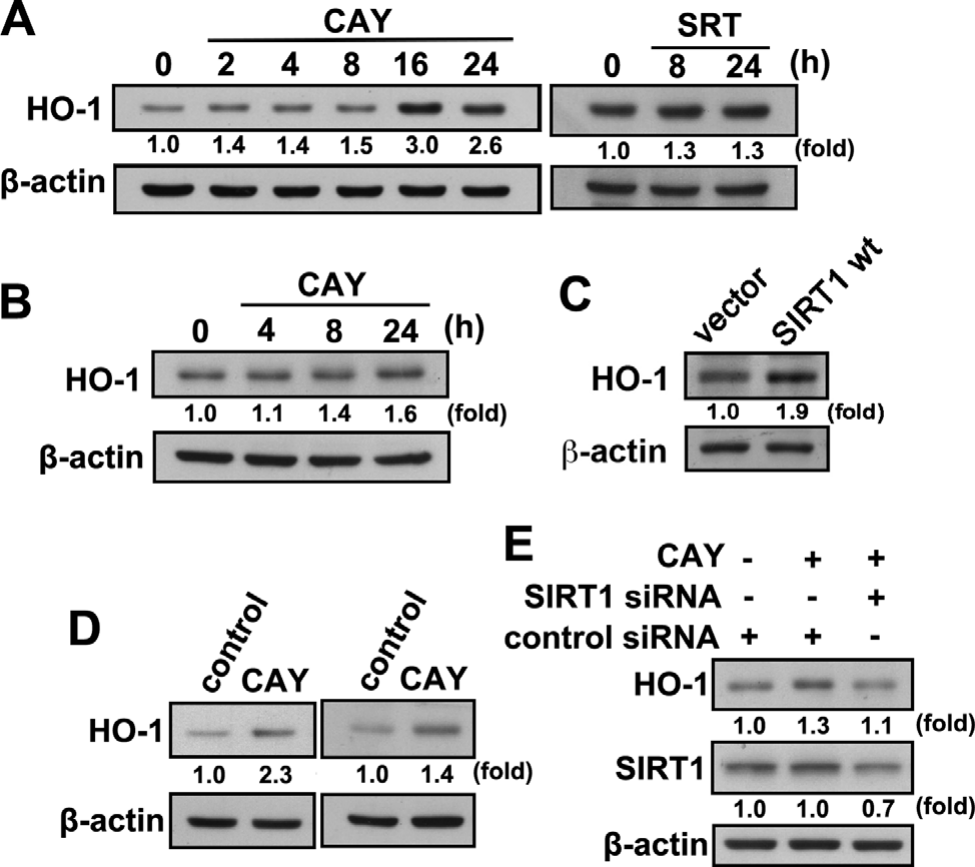
**Activation of SIRT1 induces HO-1 upregulation in microglial cells.** (**A**) BV-2 microglia were stimulated with the SIRT1 activator, CAY (5 μM) or SRT (1 μM), for the indicated time periods. (**B**) IMG cells were stimulated with the SIRT1 activator, CAY (5 μM), for the indicated time periods. (**C**) IMG cells were transfected with empty vector or wild-type SIRT1 for 24 h. (**D**) BV-2 (left panel) and IMG (right panel) microglial cells were treated with SIRT1 activator CAY (5 μM), LPS (100 ng·mL^−1^), or both CAY and LPS for 24 h. Whole-cell lysate proteins were extracted, and the HO-1 protein levels were determined by western blot analysis. (**E**) After transfection of BV-2 microglia with siRNA against SIRT1 or control for 24 h, the cells were treated with the SIRT1 activator for another 24 h. HO-1 and SIRT1 expression levels were analyzed by western blot analysis. Similar results were obtained from three independent experiments.

We have previously demonstrated that HO-1 induction is important for maintaining inflammatory homeostasis. We further investigated whether SIRT1 activation in microglia induces HO-1 expression. When microglia were treated with CAY or SRT, a time-dependent increase in HO-1 levels was observed (Figure 3A). Similarly, treatment with an SIRT1 activator increased HO-1 expression levels in adult mouse microglia (IMG; Figure 3B). Moreover, transfection with wild-type SIRT1, as compared with an empty vector, increased HO-1 expression level (Figure 3C). In addition, HO-1 expression was further enhanced by stimulation with a SIRT1 activator after LPS administration in both BV-2 and IMG cells (Figure 3D). As shown in Figure 3E, transfection with SIRT1 siRNA abrogated CAY-induced elevation in HO-1 expression level. These results suggest that SIRT1 activation induces HO-1 upregulation in microglia.

### Effects of minocycline on inflammation and SIRT1 activation in microglial cells

**Figure 4:**
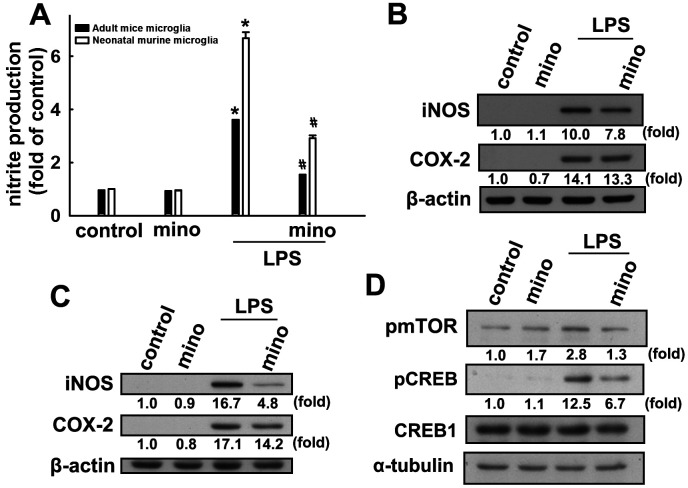
**Inhibitory effects of minocycline on inflammatory responses in microglial cells.** (**A**) Adult mice (IMG) and neonatal murine (BV-2) microglia were pretreated with minocycline (20 μM) for 30 min before stimulation with LPS (100 ng·mL^−1^) for 24 h. The cell culture medium was then harvested to determine the nitrite content by the Griess reaction. IMG (**B**) and BV-2 (**C**) microglia were pretreated with minocycline (20 μM) for 30 min before stimulation with LPS (100 ng·mL^−1^) for 24 h. Whole-cell lysates were subjected to western blot analysis for iNOS and COX-2 expression. (**D**) Cells were pretreated with minocycline (20 μM) for 30 min before stimulation with LPS (100 ng·mL^−1^) for 90 min. Whole-cell lysate proteins were subjected to western blot analysis using antibodies against phospho-mTOR or phospho-CREB. Similar results were obtained from at least three independent experiments. * *p* < 0.05, compared with the control group; # *p* < 0.05, compared with the only LPS group.

**Figure 5:**
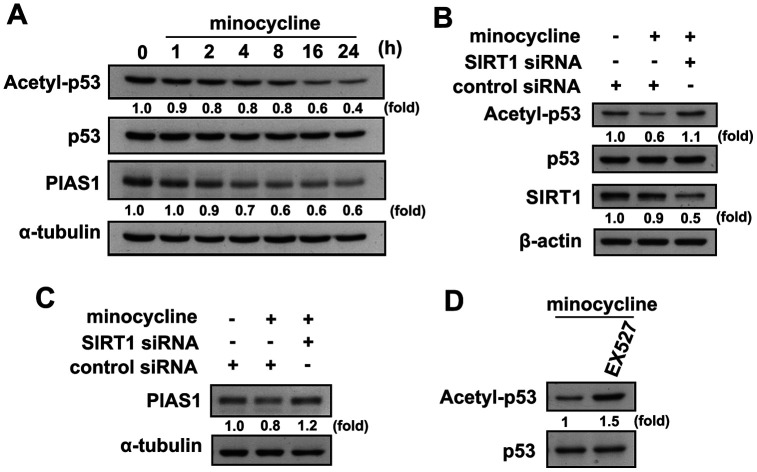
**Regulatory effects of minocycline on p53 deacetylation and PIAS1 degradation.** (**A**) BV-2 microglial cells were stimulated with minocycline (20 μM) for the indicated time periods. The expression levels of lysine 379 acetylated p53 and PIAS1 were determined by western blot analysis. (**B**, **C**) Cells were transfected with siRNA against SIRT1 or control for 24 h, and then treated with minocycline for another 24 h. (**D**) After treatment with EX527 (10 μM) for 30 min, the cells were treated with minocycline for another 24 h. Whole-cell lysate proteins were extracted and subjected to western blot analysis to assess acetyl-p53, PIAS1, and SIRT1 expression. Similar results were obtained from at least three independent experiments.

Our results support previous reports that minocycline effectively antagonizes LPS-induced nitrite production in both murine and adult mouse microglia (Figure 4A). Minocycline also effectively antagonized LPS-induced iNOS and COX-2 expressions in both murine and adult mouse microglial cells (Figure 4B and 4C). Our results also showed that LPS treatment caused a significant increase in mTOR and CREB phosphorylation in microglial cells, whereas administration of minocycline significantly abrogated LPS-enhanced protein phosphorylation (Figure 4D). We further examined whether minocycline induces p53 deacetylation and PIAS1 degradation of SIRT1 in microglial cells. As shown in Figure 5A, minocycline treatment promoted a time-dependent p53 deacetylation and PIAS1 downregulation in microglia (Figure 5A). Moreover, transfection with SIRT1 siRNA abrogated minocycline-induced p53 deacetylation (Figure 5B) and PIAS1 downregulation (Figure 5C). Moreover, treatment with EX527, a SIRT1 activity inhibitor, dramatically negated the effect of minocycline-induced p53 deacetylation (Figure 5D). These results indicate that minocycline administration induces p53 deacetylation and PIAS1 downregulation through SIRT1 activation.

### SIRT1 activation promotes microglial M2 polarization

**Figure 6:**
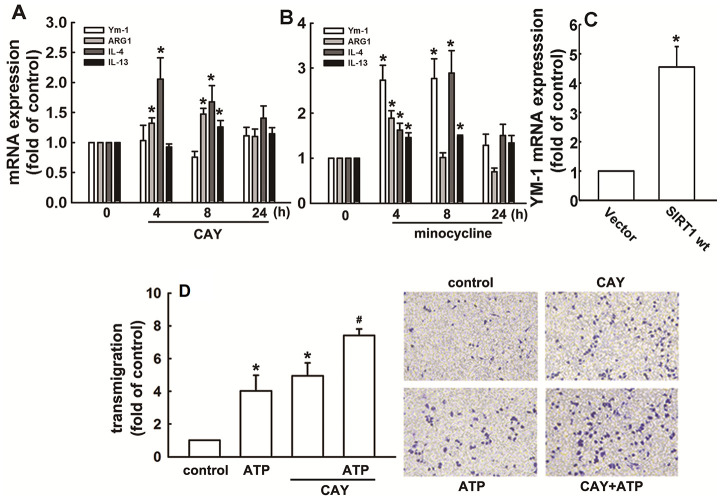
**Regulatory effects of SIRT1 on M2 polarization in microglia.** BV-2 microglia were incubated with CAY (5 μM) (**A**) or minocycline (20 μM) (**B**) for the indicated time periods. (**C**) IMG cells were transfected with wild-type SIRT1 for 24 h. The expression levels of Ym-1, ARG1, IL-4, and IL-13 were determined by real-time PCR analysis. (**D**) Cells were pretreated with CAY (5 μM) for 30 min, followed by stimulation with ATP (300 μM) or no stimulation for 24 h. Transmigration activities were examined *in vitro* using a transwell insert system. The transmigrated cells were visualized by phase-contrast imaging (right panel). Results are expressed as means ± SEM of three independent experiments; * *p* < 0.05 compared with the control group. # *p* < 0.05 compared with the only ATP- or only CAY-treated groups.

**Figure 7:**
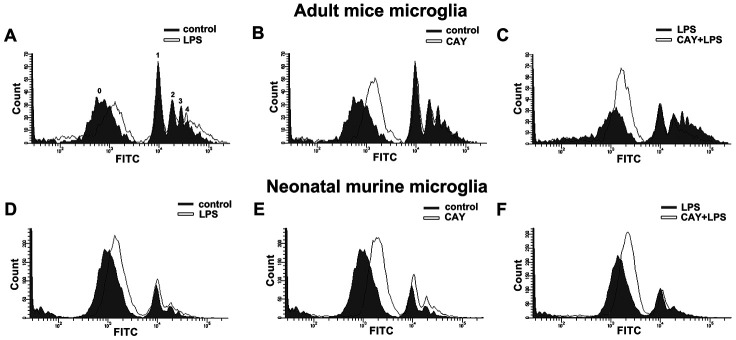
**Phagocytic activity of SIRT1 activated microglia.** Flow cytometry was used to assess the phagocytic activity by quantifying the number of 1-μm fluorescent yellow-green latex beads (YG beads) engulfed by IMG (**A**–**C**) and BV-2 (**D**–**F**) microglia. Cells with or without LPS stimulation (100 ng·mL^−1^) (**A** and **D**), treated with SIRT1 activator CAY (5 μM) (**B** and **E**), or pretreated with CAY for 30 min before 24-h LPS stimulation (**C** and **F**), were incubated with YG beads for 1 h at 37°C. Each graph represents the results from at least 3 biological replicates.

**Figure 8:**
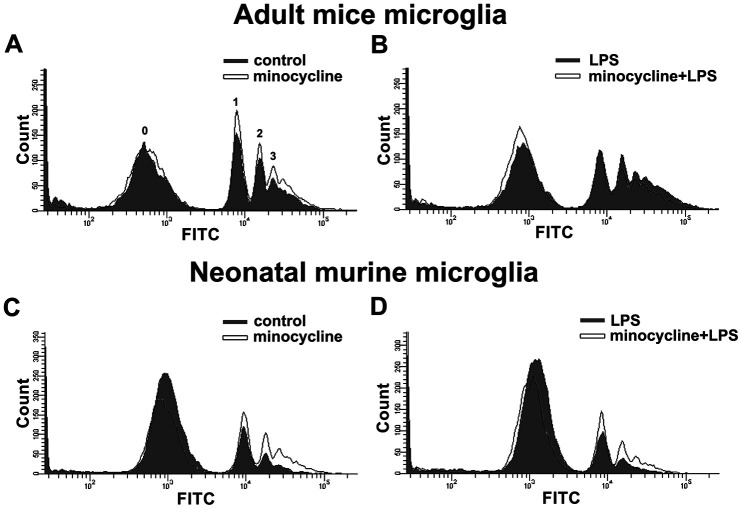
**Effects of minocycline on phagocytosis in microglia.** Phagocytosis of 1-μm fluorescent YG beads by IMG and BV-2 microglia was analyzed by flow cytometry. (**A**, **C**) Cells with or without minocycline treatment (20 μM), or (**B**, **D**) those pretreated with minocycline for 30 min before LPS stimulation for 24 h, were incubated with the YG beads for 1 h at 37°C before flow cytometry analysis. Each graph represents data from at least 3 biological replicates.

As shown in Figure 6A, SIRT1 activation increased the mRNA expression of M2 phenotype genes, including *ARG1*, *IL-4*, and *IL-13*. Furthermore, minocycline treatment increased the expressions of *IL-4*, *IL-13*, *ARG1*, and Ym-1 (Figure 6B). In addition, SIRT1 overexpression effectively increased Ym-1 expression in adult mouse microglia (Figure 6C). As shown in Figure 6D, both ATP and CAY increased the migration activity of microglial cells. Furthermore, co-treatment with CAY and ATP further increased microglial cell migration (Figure 6D). Next, we determined whether SIRT1 activation affects microglial phagocytosis. By monitoring the degree of fluorescence intensity, populations of adult mouse or neonatal murine microglia that engulfed 0, 1, 2, 3, or 4 beads can be differentiated (as labeled in Figures 7A and 8A). LPS challenge significantly increased the phagocytic activity in both adult and neonatal mouse microglia (Figure 7A and 7D). As shown in Table 1, LPS stimulation increased the percentage of cells that engulfed ≥1 YG beads and elevated the fluorescence intensity as compared with the control groups in both adult and neonatal mouse microglial cells. In adult mouse microglia, SIRT1 activation by CAY decreased the population of cells that engulfed ≥1 fluorescent bead and mildly reduced the fluorescence intensity (Figure 7B and Table 1). SIRT1 activation significantly induced microglial phagocytosis in the neonatal murine microglia (Figure 7E and Table 1). However, stimulation with a SIRT1 activator before LPS treatment dramatically attenuated LPS-induced phagocytic activity (Figure 7C), as indicated by the decreased percentage of cells and fluorescence intensity (Table 1). Moreover, treatment with an SIRT1 activator before LPS treatment in neonatal murine microglia effectively decreased the population of cells that engulfed fluorescent beads after LPS treatment and slightly reduced the fluorescence intensity as compared with the LPS group (Figure 7F and Table 1).

**Table 1:** Quantitative data from flow cytometry analysis of phagocytosis activity between adult mice microglia and neonatal murine microglia subjected to CAY and LPS treatments.

	**Adult mice microglia**		**Neonatal murine microglia**	
	**% total**	**FITC mean**	**% total**	**FITC mean**
control	39.5±1.0	20099.4±888.7	14.8±1.33	13842.5±258.2
LPS	41.3±1.6*	25809.4±186.8**	22.1±2.05*	15254.9±615.2*
CAY	32.3±1.4*	18611.5±794.4	17.5±0.92*	15831.3±467.9*
CAY+LPS	25.2±2.2^**##**^	20784.9±1021.7^**##**^	19.2±1.03^**#**^	15140.6±370.1

### Minocycline enhances phagocytic activity in microglial cells and inhibits LPS-induced inflammatory responses in a mouse model

**Figure 9:**
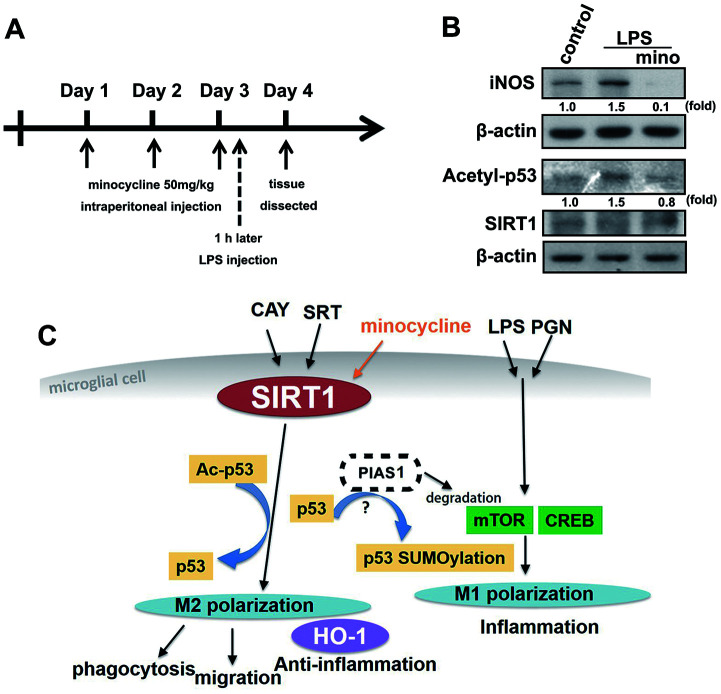
**The protective effect of minocycline prevents LPS-induced acetylation of p53 in a mouse model.** (**A**) Schematic representation of the protocol for minocycline and LPS administration. Mice were treated with either minocycline (50 mg·kg^−1^) or vehicle, once daily for three consecutive days, before a single intraperitoneal injection of LPS (20 mg·kg^−1^). LPS was administered to mice on the third day 1 h after minocycline administration. (**B**) The mice were sacrificed, and their brain cortex were dissected 1 day after LPS injection and analyzed by western blot to assess the presence of the indicated proteins. (**C**) The schema of the regulatory mechanism of minocycline on SIRT1 and p53 in microglial polarizations.

Minocycline treatment enhanced phagocytosis in both adult brain and neonatal murine microglia (Figure 8A and 8C). As shown in Table 2, minocycline administration increased the percentage of cells that engulfed ≥1 YG bead and increased the fluorescence intensity as compared with the control groups in both adult brain and neonatal murine microglial cells. As shown in Figure 8B and Table 2, minocycline pretreatment before LPS stimulation significantly attenuated LPS-induced microglial phagocytosis as indicated by the decreased cell population and low fluorescence intensity in adult mouse microglia. Unexpectedly, minocycline treatment before LPS stimulation increased the phagocytic activity in neonatal murine microglia (Figure 8D) as indicated by the increased cell population and fluorescence intensity relative to the LPS group (Table 2). To investigate the anti-inflammatory effect of minocycline *in vivo*, we analyzed changes in protein levels in tissue samples from mouse cortices. The mice were treated with minocycline (50 mg/kg) once daily for 3 consecutive days, followed by a single injection of LPS for another 24 h (Figure 9A). The mouse cortices were dissected 1 day after the LPS injection and analyzed by western blotting. As shown in Figure 9B, the LPS injection alone increased the iNOS and acetyl-p53 expression. As expected, treatment with minocycline before LPS stimulation significantly inhibited the enhancement of LPS-induced inflammatory response.

**Table 2:** Quantitative data from flow cytometry analysis of phagocytosis activity between adult mice microglia and neonatal murine microglia subjected to minocycline and LPS treatments.

	**Adult mice microglia**		**Neonatal murine microglia**	
	**% total**	**FITC mean**	**% total**	**FITC mean**
control	42 ±2.1	18785.9±234	14.6±2.6	14306.2±664.4
minocycline	46 ±2.1*	20210.6±316.1*	23 ±4.5*	16385.8±1252.9*
LPS	45.8±4.9	25308±412.2	20.5±2.8	15418 ±867.4
minocycline+LPS	41 ±5.7^**#**^	22467.3±410.5^**#**^	28.7±2.4^**#**^	17124.3±738.2^**#**^

## DISCUSSION

Our findings indicate that SIRT1 activation reduces lipopolysaccharide (LPS)- or peptidoglycan (PGN)-induced inflammatory responses that may be mediated by p53 deacetylation in microglial cells. In addition, SIRT1 activation also induces HO-1 expression and facilitates the shift of a microglial phenotype toward M2 polarization. Moreover, administration of minocycline, an inhibitor of microglial activation, suppresses microglial activation partly through SIRT1 activation. Our results also showed that minocycline decreased the iNOS and acetyl-p53 levels in the brain tissue of an inflammatory mouse model.

Currently, clinical trials are being conducted for SIRT1 activators in the treatment of age-associated diseases and type 2 diabetes [51]. The SIRT 1 activator, SRT1720, exerts a neuroprotective effect that promotes neurological recovery and striatal lesion reduction in cerebral oxidative stress [52]. In addition, SIRT1 activation reduces p53 acetylation and downstream signaling mediated by 17β-estradiol in aging mice [53]. SIRT1 activation has also offers protection against subarachnoid hemorrhage by p53 deacetylation-mediated oxidation and inflammatory response [54]. Omega-3 fatty acids activate SIRT1-mediated inhibition of NF-κB stress response, suppressing microglial neuroinflammation [55]. In addition, inhibition of SIRT1 expression reduced p53 deacetylation and promoted the activation of p53-mediated increase in the expression levels of proinflammatory genes in the mouse brain [56]. p53 deacetylation by SIRT1 attenuates p53-mediated cell death signaling and protects the brain from cerebral ischemic damage [57, 58]. In addition, Knockout of Sirt1 in brain endothelial cell affects blood brain barrier (BBB) integrity and loss increasing permeability [59]. Our present study results support previous reports that SIRT1 activation in microglial cells significantly inhibits the expression of proinflammatory cytokines such as iNOS and IL-1β, and decreases p53 acetylation.

p53 expression is also associated with neurodegenerative diseases such as AD and Parkinson’s disease (PD). p53 expression in the human brain is associated with autosomal recessive forms of juvenile PD [60]. Moreover, increased p53 expression level has been observed in microglial cells in the brains of patients with AD [32, 33], but a p53 knockout mouse model of AD displayed reduced tau phosphorylation [61]. In addition, inhibition of p53-mediated pathways attenuated microglial-triggered neurotoxicity induced by β-amyloid exposure [62]. Furthermore, inhibition of p53 expression in microglia causes less neurotoxicity in response to proinflammatory stimuli [62, 63]. Moreover, p53 deficiency promotes microglial anti-inflammatory responses [64]. Inhibition of microglial p53 activation suppresses microglial inflammatory responses and exerts a neuroprotective effect [35]. Thus, inhibition of p53-mediated transcription in microglia prevented neurotoxicity, which suggests that targeting p53-mediated pathways in microglia may have therapeutic benefits for patients with CNS injury and diseases exacerbated by inflammatory responses [65].

The M2-phenotype microglial cells strongly elicit phagocytosis of debris to prevent secondary inflammatory responses and promote tissue regeneration than the M1-phenotype microglial cells [66]. p53 deficiency in microglia increased the microglial phagocytic activity and was associated with anti-inflammatory functions [64]. SIRT1 activation alters microglial polarization, which attenuated experimental traumatic brain injury by omega-fatty acid supplementation [67]. Moreover, induction of SIRT1 expression in microglia reverses LPS-induced polarization from the M1 to M2 phenotype [68]. Furthermore, minocycline treatment enhances the phagocytosis of beta-amyloid fibrils to attenuate neuroinflammatory response [69]. Minocycline treatment also reversed the M1 response to M2 in rats subjected to chronic, unpredictable mild stress, which is associated with behavioral normalization [70]. Our results support those of previous studies which showed that both SIRT1 activation or minocycline treatment elevated the expression levels of M2 macrophage genes and phagocytic activity. In a diabetic animal model, minocycline treatment inhibited histone acetylation in retinal cells [71] which contributed to the beneficial effects of diabetic retinopathy treatment [72]. The results from this mouse model further support previous findings that minocycline-antagonized LPS-induced inflammation could be attributed to the induction of SIRT1 activity and the subsequent deacetylation of p53 by SIRT1 (Figure 9). In addition, we found some differences between adult mouse and neonatal murine microglia in their response to minocycline treatment (Figure 8B and 8D). Our results support previous report that primary adult microglia, rather than neonatal murine microglia, may share phenotypic characteristics with the novel immortalized adult microglial cell line IMG [73].

The protein inhibitor of activated stat (PIAS) 1 was initially identified as a suppressor of interferon-dependent transcription [74]. We previously reported that PIAS1 degradation is involved in microglial activation and proinflammatory expression [50]. Both PIAS1 and PIASy promote the SUMOylation and transcriptional activity of p53 [75, 76]. Moreover, controlling the activity of both p53 and PIASy regulate Ras-induced senescence and apoptosis [76]. SUMOylation of p53 contributes to endothelial inflammation and atherosclerotic plaque formation [77]. Moreover, PIAS3 overexpression blocked the HO-1 expression induced by IL-6 in HepG2 cells [78]. In this study, our observations showed that SIRT1 activation or minocycline treatment induced PIAS1 degradation in microglia. However, further study is required to identify the detailed mechanism of regulatory effect of PIAS1 on neuroinflammation.

In conclusion, SIRT1 activation inhibits LPS- or PGN-induced inflammatory responses mediated by p53 deacetylation. SIRT1 activation also facilitates microglial M2 polarization and induces HO-1 expression, PIAS1 degradation, migration, and phagocytosis of microglial cells. Therefore, our results indicate that SIRT1 activation may be a useful therapeutic target for the treatment of neuroinflammation-associated disorders owing to its function in the modulation of inflammatory homeostasis that could contribute to anti-neuroinflammatory effects. Our results also provide new insights into the effects of minocycline treatment on SIRT1 expression in the maintenance of M1/M2 polarization homeostasis of microglia (Figure 9C), which are useful to identify a novel therapeutic target for neuroinflammation-associated disorders.

## MATERIALS AND METHODS

### Materials

CAY10591 and SRT1720 were purchased from Cayman Chemicals (Ann Arbor, MI, USA). Primary antibodies against β-actin, CREB (sc-25785), and NF-κΒ p65 (sc-7151) were purchased from Santa Cruz Biotechnology (Santa Cruz, CA). Antibodies against phospho-AMPK^Thr172^ (#2535), acetyl-p53^Lys379^ (#2570), phospho-p53^Ser15^, phospho-mTOR^Ser2448^, and phospho-CREB^Ser133^ (#9198) were purchased from Cell Signaling Technology. The primary antibodies against iNOS (610431) and p53 were purchased from BD Transduction Lab (Lexington, KY, USA). The primary antibody against COX-2 (aa 570-598) was purchased from Cayman Chemicals (Ann Arbor, MI, USA). Antibodies against α-tubulin (T5168), acetylated tubulin^Lys40^ (T7451), HDAC6, EX527 (E7034), and TSA (T8552) were purchased from Sigma Aldrich (St. Louis, MO). Antibodies against HO-1 were purchased from Enzo Life Sciences, Inc. (Farmingdale, NY, USA). Antibodies against β-actin (ab6267) and SIRT1 (ab50517) were purchased from Abcam (Cambridge, MA, USA).

### Animal experimental procedures

All animal experiments were approved by the Animal Care Committee of China Medical University (Taichung, Taiwan; 2017-138). The animals were manipulated in accordance with the Animal Care and Use Guidelines of China Medical University (Taichung, Taiwan). Eight-week-old male imprinting control region (ICR) mice were purchased from the National Laboratory Animal Center (Taipei, Taiwan). The animals were housed in a temperature- and humidity-controlled environment and given access to food and water *ad libitum*. The mice were acclimated to their environment for 7 days before the experiments.

### Tissue preparation

Eight-week-old mice received intraperitoneal injections of vehicle or minocycline (50 mg·kg^−1^) once daily for three consecutive days before a single intraperitoneal injection of LPS (20 mg·kg^−1^). On the third day, 1 h after minocycline administration, LPS was administered to the mice. The cortex was collected 24 h after LPS injection.

### Microglial cells

Adult mouse microglia cell line (IMG) derived from adult brain was obtained from Harvard School of Public Health (Boston, MA, USA). IMG cells express a microglial-specific marker, fully recapitulate the morphological and functional characteristics of brain microglia, and share phenotypic attributes with primary adult microglia [73].

The neonatal murine microglial cell line, BV-2, tested positive for macrophage antigen complex (MAC)-1 and -2 antigens. Currently, BV-2 cells are used as a model system because they retain most of the morphological, phenotypical, and functional properties described for freshly isolated microglial cells [79]. BV-2 cells were cultured in Dulbecco’s modified Eagle medium with high glucose (4.5 g·L^−1^), 10% fetal bovine serum (FBS), and penicillin/streptomycin (100 U·mL^−1^) at 37°C in a humidified incubator with 5% CO_2_ and 95% air.

### Western blot analysis

Cells were treated with CAY10591 and SRT1720 for the indicated time periods, washed with cold phosphate-buffered saline (PBS), and lysed with a lysis buffer for 30 min on ice. Protein samples from the lysate were separated by sodium dodecyl sulfate polyacrylamide gel electrophoresis and blotted onto polyvinylidene fluoride membranes. The membranes were then blocked with 5% nonfat milk and probed with the relevant primary antibody. After several PBS washes, the membranes were incubated with secondary antibodies. The target proteins on the blots were visualized by enhanced chemiluminescence using Kodak X-OMAT LS film (Eastman Kodak, Rochester, NY). Quantitative data were obtained by computing the densitometric values using ImageQuant software (Molecular Dynamics, Sunnyvale, CA).

### Nitric oxide assay

Production of NO was assayed by measuring the stable nitrite levels formed as a result of nitric oxide metabolism in the culture medium, which was prepared as described previously [80].

### RNA extraction and quantitative real-time polymerase chain reaction (PCR)

Total RNA was extracted from cells using TRIzol reagent (Invitrogen, Carlsbad, CA, USA) and quantified using a BioDrop spectrophotometer (Cambridge, UK). Target mRNA levels were detected using quantitative real-time PCR. The reverse transcription (RT) reaction was performed using 2 μg of total RNA that was converted to cDNA using the Invitrogen RT Kit and amplified using the oligonucleotide primers. The threshold was set within the linear phase of target gene amplification to calculate the cycle number at which the transcript was detected (denoted as CT).

### Cell transfection

Small interfering RNA (siRNA) si-GE-NOME duplexes targeting mouse SIRT1 were acquired from Dharmacon, and microglia were transfected with 2 μL of DharmaFECT Reagent #1 with 20 nM si-GENOME siRNA (Dharmacon/Thermoscientific). The microglia were seeded in a six-well plate at 70% confluency 24 h before transfection. The mixture of siRNA and DharmaFECT was added to 1.6 mL complete microglia growth media supplemented with 10% FBS and added to the cells. The cells were incubated with the transfection mixture for 48 h before the experiment.

### Transmigration assay

Transmigration assays were performed using Costar Transwell inserts (Costar, NY; pore size, 8 μm) in 24-well plates as described previously [64]. Approximately 1 × 10^4^ cells in 200 μL of serum-free medium were placed in the upper chamber, and 300 μL of the same medium containing ATP was placed in the lower chamber. The plates were incubated for 24 h at 37°C in 5% CO_2_, and then the cells were stained with 0.05% crystal violet and 2% methanol. Non-migrating cells on the upper surface of the filters were removed by scraping with a cotton swab. The cell number in three fields of each well was counted under a microscope at 100× magnification. Images of the migrating cells were observed and acquired using a digital camera and light microscope.

### Phagocytosis assay

Cells were incubated with carboxylate-modified polystyrene fluorescent yellow-green latex beads (YG beads; Cat# L4655; Sigma Aldrich) at 37°C to permit phagocytosis. After exposure to YG beads, microglial cells were washed thoroughly and analyzed for yellow-green fluorescence by flow cytometry. The cells were seeded into four 6-well tissue culture plates (5 × 10^5^ cells/well). They were allowed to grow for 16 h at 37°C/5% CO_2_, followed by treatment with SIRT1 activator or LPS for another 24 h before the assay. Eighty-five μL) of YG beads were diluted in 8.5 mL aliquots of pre-warmed (37°C) growth media. Media was removed from the cells and 1 mL of YG bead-containing media was added to each well. The cells were immediately incubated at 37°C for 1 h. The remaining steps were strictly performed on ice. To remove non-internalized beads, the cells were washed with PBS. After washing, the cells were incubated with PBS containing 2 mM ethylenediaminetetraacetic acid (EDTA) for 10 min at 4°C. Thereafter, the cells were removed from the dish by trypsin treatment and transferred to 1.5 mL Eppendorf vials. They were centrifuged at 300 × g for 6 min at 4°C. The supernatant was discarded and the cell pellets were washed twice with ice-cold PBS and resuspended in PBS containing 2 mM EDTA. The cell that phagocytosed the YG beads were quantified by flow cytometry.

### Statistical analysis

Statistical analysis was performed using GraphPad Prism 6.0 (Graph Pad Software Inc, San Diego, CA, USA). The values are presented as mean ± standard error of the mean. The significance of the difference between the experimental group and control groups was assessed using Student’s *t-*test. Statistical comparisons of more than 2 groups were performed using one-way analysis of variance with Bonferroni post hoc test. Differences were considered to be significant if the *p-*value was <0.05.
